# Supplementary material for: Role of UeMsb2 in Filamentous Growth and Pathogenicity of Ustilago esculenta
Source: J Fungi (Basel). 2024 Nov 25;10(12):818. doi: 10.3390/jof10120818 (PMC11677758; doi:10.3390/jof10120818)
Supplement: Supplementary file 1 [file jof-10-00818-s001.zip › jof-3324815-supplementary/Figure S2.pdf]

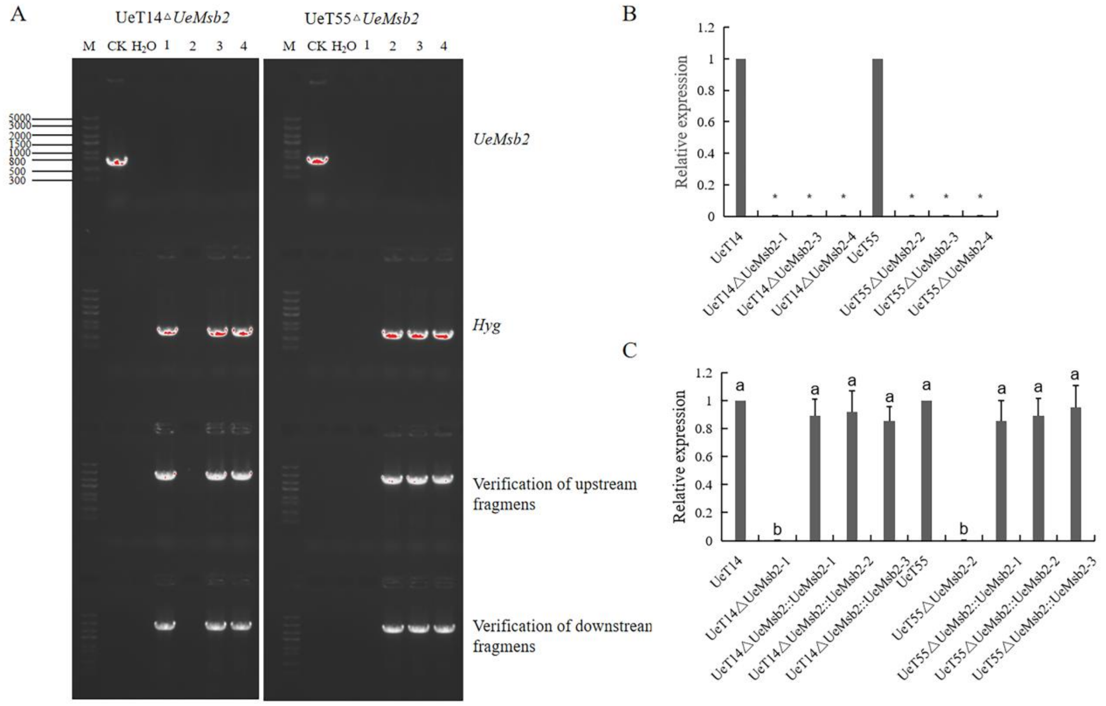

Figure S2. Validation of *UeMsb2* deletion strains and complementation strains. (A) PCR validation of *UeMsb2* deletion strain: Lanes 1, 3 and 4 of UeT14△*UeMsb2* and lanes 2 ,3 and 4 of UeT55△*UeMsb2* was identified as a positive transformants. M: Trans5k DNA marker; CK and H_2O are positive and negative controls, respectively. (B) qRT-PCR validation of deletion strain, with *β-actin* as the internal reference gene. The relative expression level of *UeMsb2* in UeT14 and UeT55 was set to '1', and One-way ANOVA was used to analyze the data, n=3. * indicating a significant difference in the expression level of *UeMsb2* between the strain and the positive control ($p<0.05$). (C) qRT-PCR validation of complementation strain, with *β-actin* as the internal reference gene. Common comparison of expression levels between deletion strain and complementation strain. The relative expression level of *UeMsb2* in UeT14 and UeT55 was set to '1', and One-way ANOVA was used to analyze the data, n=3; Different lowercase letters indicate significant difference ($P<0.05$). The expression of *UeMsb2* gene in the corresponding complementation strain was significantly different from that in the deletion strain ($p<0.05$), but was not significantly different from that in the wild-type strain UeT14 and UeT55.
