# Supplementary material for: Role of UeMsb2 in Filamentous Growth and Pathogenicity of Ustilago esculenta
Source: J Fungi (Basel). 2024 Nov 25;10(12):818. doi: 10.3390/jof10120818 (PMC11677758; doi:10.3390/jof10120818)
Supplement: Supplementary file 1 [file jof-10-00818-s001.zip › jof-3324815-supplementary/Figure S3.pdf]

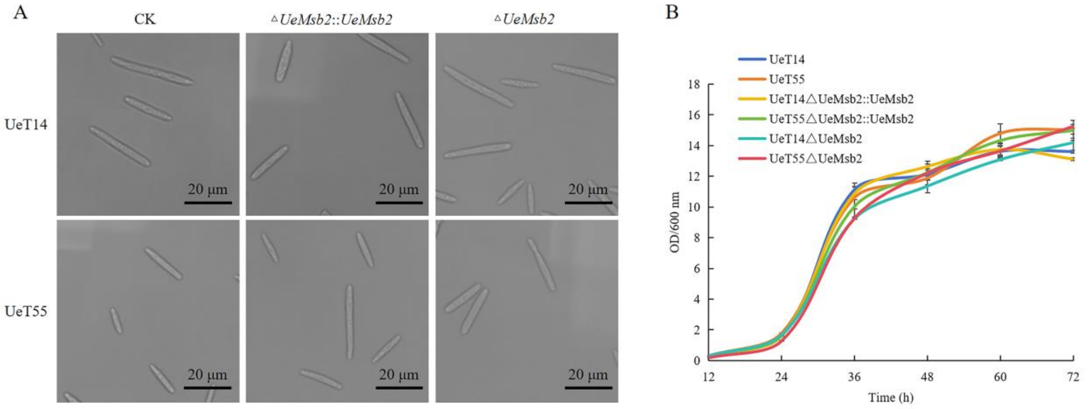

Figure S3. The deletion of *UeMsb2* did not affect the cell morphology (A) and growth rate (B) of *U. esculenta*. (A) Left: UeT14 (top) and UeT55 (bottom), as controls; Middle: UeT14△*UeMsb2*::*UeMsb2* (top) and UeT55△*UeMsb2*::*UeMsb2* (bottom); Right: UeT14△*UeMsb2* (top) and UeT55△*UeMsb2* (bottom).
