# Supplementary material for: Role of UeMsb2 in Filamentous Growth and Pathogenicity of Ustilago esculenta
Source: J Fungi (Basel). 2024 Nov 25;10(12):818. doi: 10.3390/jof10120818 (PMC11677758; doi:10.3390/jof10120818)
Supplement: Supplementary file 1 [file jof-10-00818-s001.zip › jof-3324815-supplementary/Figure S4.pdf]

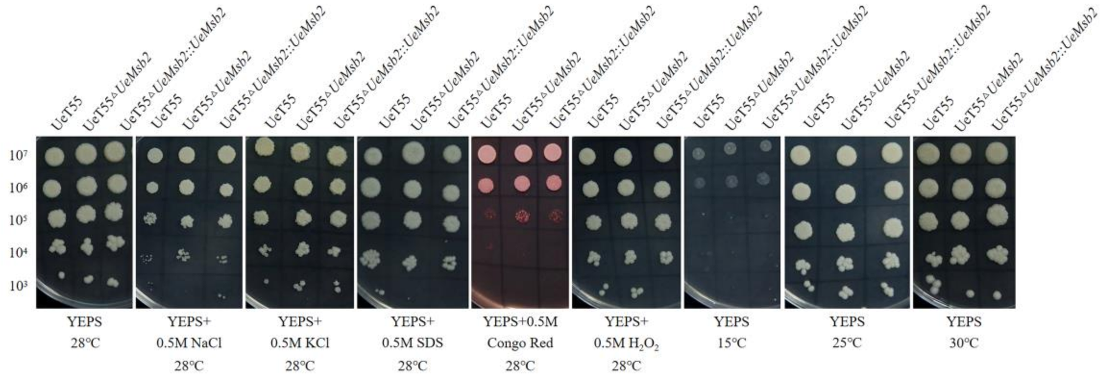

Figure S4. The deletion of *UeMsb2* did not affect the growth of haploid *U. esculenta* strains under different stress conditions.
