# Supplementary material for: Role of UeMsb2 in Filamentous Growth and Pathogenicity of Ustilago esculenta
Source: J Fungi (Basel). 2024 Nov 25;10(12):818. doi: 10.3390/jof10120818 (PMC11677758; doi:10.3390/jof10120818)
Supplement: Supplementary file 1 [file Figure S5.pdf › jof-3324815-supplementary/Figure S5.pdf]

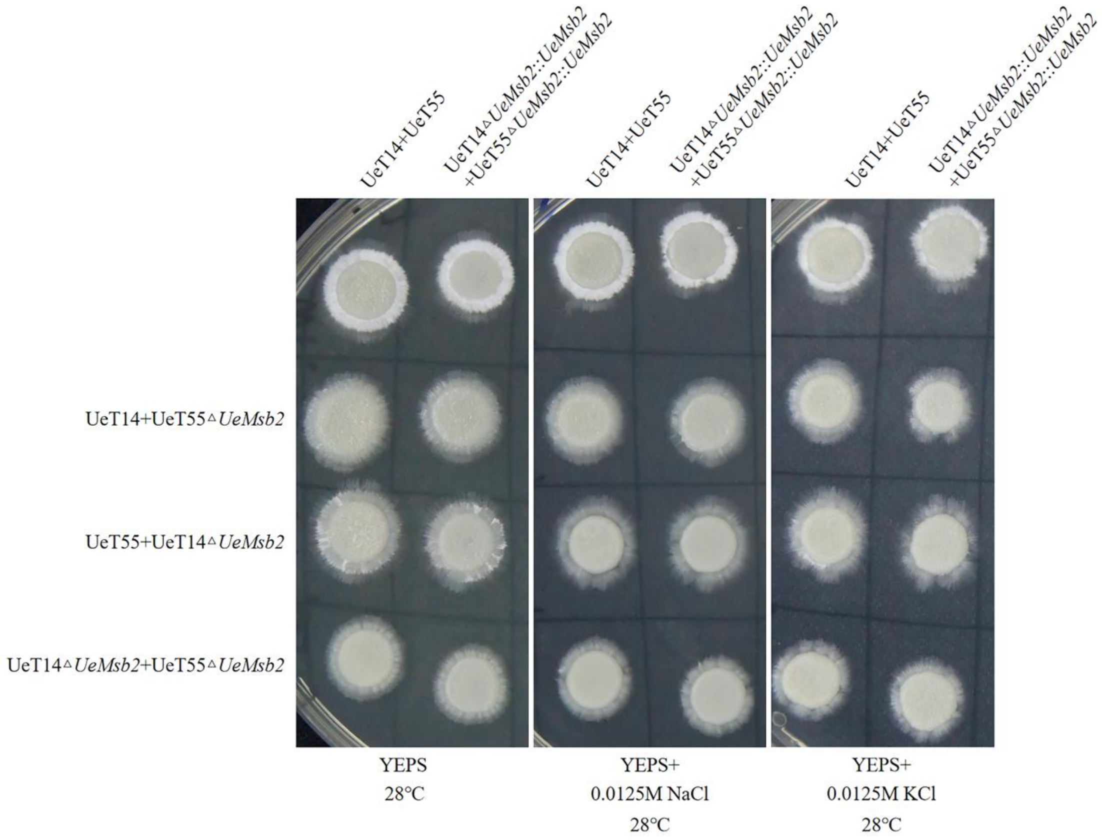

Figure S5. The deletion of *UeMsb2* did not affect the cell fusion in vitro under high osmotic stress. Colony photographs were taken 72 hours after fusion. The fusion colonies on the left side, from top to bottom, are UeT14+UeT55, UeT14+UeT55△*UeMsb2*, UeT55+UeT14△*UeMsb2* and UeT14△*UeMsb2*+UeT55△*UeMsb2*; On the right side, from top to bottom, are UeT14△*UeMsb2*::*UeMsb2*+UeT55△*UeMsb2*::*UeMsb2*, UeT14+UeT55△*UeMsb2*, UeT55+UeT14△*UeMsb2*, UeT14△*UeMsb2*+UeT55△*UeMsb2*.
